# Supplementary material for: Tackling antibiotic resistance by inducing transient and robust collateral sensitivity
Source: Nat Commun. 2023 Mar 30;14:1723. doi: 10.1038/s41467-023-37357-4 (PMC10063638; doi:10.1038/s41467-023-37357-4)
Supplement: Supplementary file 1 — Supplementary Material [file 41467_2023_37357_MOESM1_ESM.pdf]

Table S1. ***Pseudomonas aeruginosa*** **PA14 antibiotic-resistant mutants used in this work.**

Mutant	Gene	Type of genetic modification	Aminoacid change
parR87	*parR*	SNP	Glu87Lys
orfN50	*orfN*	deletion	Val50fs
nfxB177	*nfxB*	SNP	Phe177Ser
mexZ43	*mexZ*	SNP	Val43Gly
MDR6	*mexC*	SNP	Thr267Ala
	pmrB	SNP	Leu87Gln
	frr	SNP	Ile98Ser
	phoQ	SNP	Val260Gly
MDR12	*fusA*	SNP	Tyr552Cys
	fusA	SNP	Tyr683Cys
	orfN	deletion	Val50fs
	pmrB	SNP	Met46Ile
	mexZ	SNP	Val43Gly
	gabP	SNP	Ser267Phe
	ptsP	SNP	Leu537Pro
	nuoC	SNP	Gln184*

*: stop. Fs: frameshift

Table S2. FIC index of the combination dequalinium chloride-tobramycin for *P. aeruginosa* PA14 and the resistant mutants used in this work.

	FIC index $_{DC\text{-}TOB}$
PA14	0.6
parR87	0.6
orfN50	0.6
mexZ43	0.35
nfxB177	0.6
MDR6	0.6
MDR12	0.35

DC: dequalinium chloride. TOB: tobramycin.

Table S3. MIC values (µg/mL) of antibiotics from different structural families in the final populations of *P. aeruginosa* after 4 days of ALE in the presence of 10 µg/mL of dequalinium chloride and in their parental strains.

		CIP	TOB	CAZ	ATM	IPM	TET	FOF	ERY
Parental	PA14	0.064	1	1	2	0.75	32	32	256
	parR87	0.19	2	1	3	1	24	32	>256
	orfN50	0.125	3	3	8	1	32	8	>256
	nfxB177	2	0.5	1	2	0.5	32	24	>256
	mexZ43	0.19	2	1	2	1	16	32	>256
	MDR6	0.125	2	1	1	0.5	24	24	>256
	MDR12	0.38	48	1	2	0.75	16	32	>256
DC ALE	PA14 1	0.064	1	1.5	2	0.75	32	32	256
	PA14 2	0.094	1	1	2	0.75	32	32	192
	PA14 3	0.094	0.75	1.5	2	1	32	24	256
	PA14 4	0.064	0.75	1	2	1	32	32	192
	parR87 1	0.25	1.5	1	3	1	24	32	>256
	parR87 2	0.25	1.5	1	3	0.75	24	32	>256
	parR87 3	0.25	1.5	1	3	1	24	32	>256
	parR87 4	0.25	1.5	1	3	1	24	32	>256
	orfN50 1	0.19	2	3	8	1	32	6	>256
	orfN50 2	0.19	2	3	8	1	32	8	>256

orfN50 3	0.19	2	3	8	1	32	8	>256
orfN50 4	0.19	2	3	6	1	32	8	>256
nfxB177 1	2	0.75	1	3	0.75	24	24	>256
nfxB177 2	2	0.75	1	3	0.5	32	24	>256
nfxB177 3	2	0.75	1	2	0.5	24	16	>256
nfxB177 4	2	0.75	1	2	0.5	24	24	192
mexZ43 1	0.25	1.5	0.75	2	1	16	32	>256
mexZ43 2	0.19	1.5	1	1.5	1	24	32	>256
mexZ43 3	0.19	1.5	1	1.5	0.75	16	24	>256
mexZ43 4	0.25	1.5	1	1.5	0.75	16	32	>256
MDR6 1	0.19	2	0.75	1	0.38	24	24	>256
MDR6 2	0.19	1.5	1	1	0.38	32	32	>256
MDR6 3	0.19	1.5	1	1	0.38	24	24	>256
MDR6 4	0.19	2	1	1	0.5	24	24	>256
MDR12 1	0.38	48	1	2	0.75	16	48	>256
MDR12 2	0.38	48	0.75	2	0.75	12	48	>256
MDR12 3	0.38	48	1	3	0.5	16	48	>256
MDR12 4	0.38	48	1	2	0.5	16	48	>256

DC: dequalinium chloride, CIP: ciprofloxacin, TOB: tobramycin. CAZ: ceftazidime, ATM: aztreonam, IPM: imipenem, TET: tetracycline, FOF: fosfomycin, ERY; erythromycin.

Table S4. MIC values (µg/mL) of ciprofloxacin and tobramycin in the final populations of *P. aeruginosa* after 15 days of ALE in the presence of 10 µg/mL of dequalinium chloride and in their parental strains.

		CIP	TOB
Parental	PA14	0.064	1
	parR87	0.19	2
	orfN50	0.125	3
	nfxB177	2	0.5
	mexZ43	0.19	2
	MDR6	0.125	2
	MDR12	0.38	48
DC ALE	PA14 1	0.094	1
	PA14 2	0.094	1
	PA14 3	0.094	1
	PA14 4	0.094	1
	parR87 1	0.19	1.5
	parR87 2	0.19	1.5
	parR87 3	0.19	2
	parR87 4	0.19	2
	orfN50 1	0.19	3
	orfN50 2	0.125	2
	orfN50 3	0.125	3
	orfN50 4	0.125	3
	nfxB177 1	0.125	0.5
	nfxB177 2	0.094	0.75
	nfxB177 3	0.125	0.5
	nfxB177 4	0.094	0.5
	mexZ43 1	0.25	2
	mexZ43 2	0.25	1.5
	mexZ43 3	0.19	2
	mexZ43 4	0.25	1.5
	MDR6 1	0.125	2
	MDR6 2	0.125	2

MDR6 3	0.125	2
MDR6 4	0.125	2
MDR12 1	0.38	12
MDR12 2	0.38	12
MDR12 3	0.25	12
MDR12 4	0.25	16

DC: dequalinium chloride, CIP: ciprofloxacin, TOB: tobramycin.

Table S5. MIC values (µg/mL) of tobramycin in the final populations of *P. aeruginosa* after 15 days of ALE in the presence of 10 µg/mL of dequalinium chloride determined in the presence and absence of dequalinium chloride.

		TOB	TOB + DC
Parental	PA14	1	0.5
	parR87	2	0.5
	orfN50	3	0.75
	nfxB177	0.5	0.5
	mexZ43	2	0.75
	MDR6	2	0.75
	MDR12	48	8
DC ALE	PA14 1	1	0.5
	PA14 2	1	0.5
	PA14 3	1	0.5
	PA14 4	1	0.5
	parR87 1	1.5	0.5
	parR87 2	1.5	0.5
	parR87 3	2	0.75
	parR87 4	2	0.75
	orfN50 1	3	1
	orfN50 2	3	1
	orfN50 3	3	1
	orfN50 4	3	0.5
	nfxB177 1	0.5	0.5
	nfxB177 2	0.75	0.5
	nfxB177 3	0.5	0.5
	nfxB177 4	0.5	0.5
	mexZ43 1	2	0.75
	mexZ43 2	1.5	0.75
	mexZ43 3	2	0.5
	mexZ43 4	1.5	0.75
	MDR6 1	2	0.75
	MDR6 2	2	1

MDR6 3	2	1
MDR6 4	2	1
MDR12 1	12	6
MDR12 2	12	6
MDR12 3	12	6
MDR12 4	16	6

DC: dequalinium chloride, TOB: tobramycin.

Table S6. Clinical isolates used in this study.

Isolate ID	Sample origin	ST	Hypermutator	MIC (mg/L)		Mutational resistome
				CIP	TOB	
CAN01-002	Sputum	111	No	0.032	1	*mexB* (Q319X), *mexY* (G530S), *mexZ* (Q140K), *mexT* (G276D), *oprD* (nt174Δ11), *mexS* (nt300IS)
MAD04-002	Sputum	242	No	0.125	1.5	*parC* (K726R)
CAT09-004	Blood	244	No	0.064	0.75	
FQSE15-0803	CF sputum	274	Yes, *mutS*(nt814Δ4)	0.19	1.5	*mexA* (L338P), *mexZ* (A144V)
FQSE11-0603	CF sputum	701	No	0.094	1.5	*mexB* (nt775Δ1), *mexY* (N709H, A586T), *mexX* (A38P), *oprN* (R363H), *ampDh2* (P116S)
ARA03-004	Sputum	845	No	0.75	2	*gyrA* (D87N), *ampD* (Q131X), *mexT* (G246S), *mexB* (nt54Δ1), *mexZ* (nt302Δ9), *dacB* (nt781Δ1)
CLE03-006	Blood	1337	No	1	2	*parR* (aa214Δ1), *gyrA* (D87N), *ampD* (D59E)
NAV01-005	Sputum	1637	No	0.38	8	*gyrB* (P749S), *mexB* (P190L), *oprD* (nt742Δ1), *mexZ* (R104W), *galU* (F248S), *fusA1* (Y552C), *mexD* (L1027V), *mexC* (V367A), *pmrB* (A173T), *armZ* (A262S)
MAD05-008	Sputum	1717	No	0.38	3	*parS* (V152A), *mexY* (T238I)
BAL04-002	Blood	1816	No	0.064	0.75	*mexA* (K86E), *ampR* (G295R), *ampC* (A278G), *parE* (E215Q)

CF: cystic fibrosis, CIP: ciprofloxacin, TOB: tobramycin. Δ: deletion. IS: insertion sequence. All the clinical isolates used in this study belong to different Spanish hospitals and were previously used in a collaborative study from our laboratory (1).

Table S7. MIC values (µg/mL) of ciprofloxacin and tobramycin in absence (-) or presence (+) of 10 µg/mL of dequalinium chloride in different *P. aeruginosa* clinical strains.

		CIP	TOB
-DC	FQSE15-0803	0.19	1.5
	CLE03-006	1	2
	MAD04-002	0.125	1.5
	MAD05-008	0.38	3
	FQSE11-0603	0.094	1.5
	BAL04-002	0.064	0.75
	CAT09-004	0.064	0.75
	NAV01-005	0.38	8
	CAN01-002	0.032	1
	ARA03-004	0.75	2
+DC	FQSE15-0803	0.38	0.38
	CLE03-006	3	0.38
	MAD04-002	0.38	0.38
	MAD05-008	1	0.38
	FQSE11-0603	0.25	0.5
	BAL04-002	0.25	0.19
	CAT09-004	0.25	0.25
	NAV01-005	0.75	1
	CAN01-002	0.19	0.19
	ARA03-004	1.5	0.38

DC: dequalinium chloride, CIP: ciprofloxacin, TOB: tobramycin.

REFERENCES

1. Hernando-Amado S, Lopez-Causape C, Laborda P, Sanz-Garcia F, Oliver A, Martinez JL. Rapid Phenotypic Convergence towards Collateral Sensitivity in Clinical Isolates of Pseudomonas aeruginosa Presenting Different Genomic Backgrounds. Microbiol Spectr. 2022:e0227622.
