# Supplementary material for: Tackling antibiotic resistance by inducing transient and robust collateral sensitivity
Source: Nat Commun. 2023 Mar 30;14:1723. doi: 10.1038/s41467-023-37357-4 (PMC10063638; doi:10.1038/s41467-023-37357-4)
Supplement: Supplementary file 3 — Reporting Summary [file 41467_2023_37357_MOESM3_ESM.pdf]

Corresponding author(s): Sara Hernando Amado

Last updated by author(s): Mar 8, 2023

Reporting Summary

Nature Portfolio wishes to improve the reproducibility of the work that we publish. This form provides structure for consistency and transparency in reporting. For further information on Nature Portfolio policies, see our Editorial Policies and the Editorial Policy Checklist.

Statistics

For all statistical analyses, confirm that the following items are present in the figure legend, table legend, main text, or Methods section.

n/a	Confirmed	
☒	☐	The exact sample size (*n*) for each experimental group/condition, given as a discrete number and unit of measurement
☒	☐	A statement on whether measurements were taken from distinct samples or whether the same sample was measured repeatedly
☒	☐	The statistical test(s) used AND whether they are one- or two-sided<br>*Only common tests should be described solely by name; describe more complex techniques in the Methods section.*
☒	☐	A description of all covariates tested
☒	☐	A description of any assumptions or corrections, such as tests of normality and adjustment for multiple comparisons
☒	☐	A full description of the statistical parameters including central tendency (e.g. means) or other basic estimates (e.g. regression coefficient) AND variation (e.g. standard deviation) or associated estimates of uncertainty (e.g. confidence intervals)
☒	☐	For null hypothesis testing, the test statistic (e.g. *F*, *t*, *r*) with confidence intervals, effect sizes, degrees of freedom and *P* value noted<br>*Give P values as exact values whenever suitable.*
☒	☐	For Bayesian analysis, information on the choice of priors and Markov chain Monte Carlo settings
☒	☐	For hierarchical and complex designs, identification of the appropriate level for tests and full reporting of outcomes
☒	☐	Estimates of effect sizes (e.g. Cohen's *d*, Pearson's *r*), indicating how they were calculated

Our web collection on statistics for biologists contains articles on many of the points above.

Software and code

Policy information about availability of computer code

Data collection	No custom software was used.
Data analysis	No custom software was used.

For manuscripts utilizing custom algorithms or software that are central to the research but not yet described in published literature, software must be made available to editors and reviewers. We strongly encourage code deposition in a community repository (e.g. GitHub). See the Nature Portfolio guidelines for submitting code & software for further information.

Data

Policy information about availability of data

All manuscripts must include a data availability statement. This statement should provide the following information, where applicable:

- Accession codes, unique identifiers, or web links for publicly available datasets
- A description of any restrictions on data availability
- For clinical datasets or third party data, please ensure that the statement adheres to our policy

All data needed to evaluate the conclusions of this work are present in the main document and/or the Supplementary Materials.

Human research participants

Policy information about studies involving human research participants and Sex and Gender in Research.

Reporting on sex and gender	Sex and gender were not relevant and, therefore, not considered in this study.
Population characteristics	The study did not include human samples.
Recruitment	The study did not include human samples.
Ethics oversight	The study did not include human or animal samples; no ethics issues were needed to be addressed.

Note that full information on the approval of the study protocol must also be provided in the manuscript.

Field-specific reporting

Please select the one below that is the best fit for your research. If you are not sure, read the appropriate sections before making your selection.

☒ Life sciences ☐ Behavioural & social sciences ☐ Ecological, evolutionary & environmental sciences

For a reference copy of the document with all sections, see nature.com/documents/nr-reporting-summary-flat.pdf

Life sciences study design

All studies must disclose on these points even when the disclosure is negative.

Sample size	We used the model strain of Pseudomonas aeruginosa PA14, six different resistant isogenic mutants constructed in the lab and ten clinical isolates from different Spanish hospitals. We included different genetic backgrounds, different mutational resistomes and some of them being high-risk epidemic clones. Sample size was the one that were previously used in different ALE assays to find robust patterns of evolution, being these genetic contexts and number of replicate populations sufficient to carry out this type of study.
Data exclusions	No data were excluded.
Replication	For ALE studies four replicate populations of each genetic background were analyzed in each specific condition, including the same number of control populations. No average values of MICs from technical replicates were determined since, by definition, MIC is the minimal concentration that inhibits growth of a specific strain. These values were independently determined -at least three times- and results were always consistent.
Randomization	Strains were specifically chosen with the purpose of having representative isolates from different genetic backgrounds and mutational resistomes. The reason is that it is known that AR mutations may lead to a robust or variable CS patterns depending on whether they produce “target” or “regulatory” alterations, respectively. That is why we included this varied set of mutants, including clinical strains.
Blinding	In general we first annotated the results and afterwards looked at the label of the plate/tube to reduce the potential bias.

Reporting for specific materials, systems and methods

We require information from authors about some types of materials, experimental systems and methods used in many studies. Here, indicate whether each material, system or method listed is relevant to your study. If you are not sure if a list item applies to your research, read the appropriate section before selecting a response.

Materials & experimental systems

n/a	Involved in the study	
☒	☐	Antibodies
☒	☐	Eukaryotic cell lines
☒	☐	Palaeontology and archaeology
☒	☐	Animals and other organisms
☒	☐	Clinical data
☒	☐	Dual use research of concern

Methods

n/a	Involved in the study	
☒	☐	ChIP-seq
☒	☐	Flow cytometry
☒	☐	MRI-based neuroimaging
